# PESTICIDES: Double Exposure Heightens Parkinson Disease Risk

**DOI:** 10.1289/ehp.117-a295

**Published:** 2009-07

**Authors:** Cynthia Washam

**Affiliations:** **Cynthia Washam** writes for *EHP, Oncology Times*, and other science and medical publications from South Florida

Approximately 1 million U.S. residents and more than 4 million people worldwide have been diagnosed with Parkinson disease (PD), a chronic motor system disorder that usually strikes people over age 60 and triples in those over 85. Epidemiologic studies have consistently found that pesticide exposures heighten the risk of developing PD, a connection further strengthened by rodent studies. However, most studies to date have relied on self-reports and recall of chemical usage, bias-prone mechanisms that make it hard to accurately estimate exposure. A team of researchers led by epidemiologist Beate Ritz of the University of California, Los Angeles (UCLA) may have overcome that hurdle with a new exposure model.

Their study, published 15 April 2009 in the *American Journal of Epidemiology*, suggests that two commonly used pesticides increase PD risk, especially in people exposed at an early age. The UCLA team focused on the combined effects of the herbicide paraquat and the fungicide maneb. When used either alone or in combination with maneb, paraquat had been found in several rodent studies to cause neuronal degeneration and symptoms like those seen in PD patients.

The researchers compared pesticide exposures for 368 California residents diagnosed with PD between January 1998 and January 2007 and 341 randomly selected local controls. The subjects lived in agricultural regions in Fresno, Tulare, and Kern counties; most were white, over age 60, and had no family history of PD.

Exposure estimates came from maps of land use and from a geographic information system incorporating pesticide application records from the California Department of Pesticide Regulation. “We found a way to assess historic pesticide exposure without relying on subject recall,” says first author Sadie Costello, now a postdoctoral research fellow in the Department of Environmental Health Sciences at the University of California, Berkeley, School of Public Health. Both data sets were linked to subjects’ current and former addresses within the three-county region.

The researchers examined subjects’ exposure from 1974 to 1989, from 1990 to 1999, and for the entire time period. Subjects were considered exposed if one or both pesticides were applied within 500 m of their homes while they lived there. Exposure to both pesticides during the 25-year period was associated with a 75% increase in PD risk.

Yet associations varied depending on subjects’ age and the timing of exposure. In participants aged 60 or younger at diagnosis (cases) or interview (controls), estimated relative risks of PD with exposure to one or both pesticides were similar regardless of whether exposure occurred between 1974 and 1989 or 1990 and 1999. However, among participants over 60 years of age, associations with PD appeared to be limited to pesticide exposures during the earlier (1974 –1989) time period.

Freya Kamel, an NIEHS epidemiologist who has studied the suspected link between pesticides and PD, cautions against assuming that age at exposure can explain the different PD risks. “I don’t think I’d want to speculate until I saw it in more studies,” she says. She notes that the basic precept that developing brains are more vulnerable to neurotoxicants usually applies to prenatal and infant exposures, not to exposures in childhood or young adulthood.

Kamel predicts future studies will move toward determining how genetics affects individuals’ susceptibility to environmental toxicants. “Assessing gene–pesticide interactions is a goal of our research,” Costello says. “We have a couple of papers on the topic coming out shortly.”

